# Supplementary material for: Multivariate Analysis Reveals That Unsubstituted β-Ring and C8-Keto Structures Are Important Factors for Anti-Inflammatory Activity of Carotenoids
Source: Nutrients. 2021 Oct 21;13(11):3699. doi: 10.3390/nu13113699 (PMC8622589; doi:10.3390/nu13113699)
Supplement: Supplementary file 1 [file nutrients-13-03699-s001.zip › nutrients-1393425-supplementary.pdf]

Supplementary Information

Multivariate Analysis Reveals that Unsubstituted β-Ring and C8-Keto Structures are Important Factors for Anti-Inflammatory Activity of Carotenoids

Yuki Manabe, Nami Tomonaga, Takashi Maoka and Tatsuya Sugawara

TABLE OF CONTENTS

Figure S1. Effects of 0.5 and 1.0 μM of siphonaxanthin on the activity of NF-κB in AGE-BSA-, LPS-, or TNF-α-stimulated THP1-Dual cells

Figure S2. Effects of antagonism of RAR on suppression of TNF-α-NF-κB axis and promotion of PMA-IRF axis by β-carotene

Figure S3. LC-MS/MS analysis of retinoids

Figure S4. HPLC-PDA analysis of siphonaxanthin and its metabolites

Table S1. The parameters for the MRM detection in LC-MS analysis of carotenoids and retinoids.

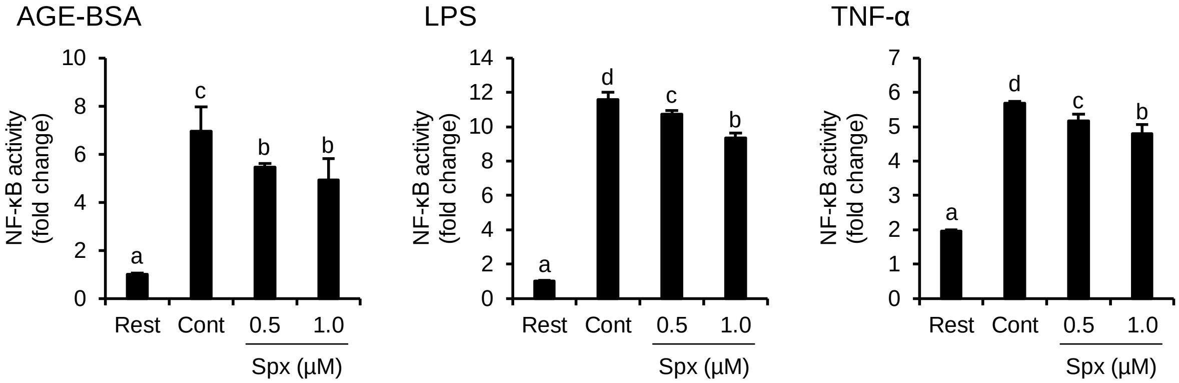

Figure S1. Effects of 0.5 and 1.0 μM of siphonaxanthin on the activity of NF-κB in AGE-BSA-, LPS-, or TNF-α-stimulated THP1-Dual cells. THP1-Dual cells were incubated with 0.5 or 1.0 μM of siphonaxanthin, followed by stimulation with the indicated agent. Siphonaxanthin was dispersed in culture medium using DMSO (maximum final concentration, 0.1%) as a vehicle. The resting cell and control groups were incubated for 24 h, followed by no stimulation or stimulation with inflammatory inducer, respectively. After stimulation, SEAP activities in the supernatant were measured ($n = 4$). Values represent mean ± SD. Significant differences were detected by one-way ANOVA in AGE-BSA ($F(3,12) = 55.0$, $p < 0.05$), LPS ($F(3,12) = 1317$, $p < 0.05$), and TNF-α ($F(3,12) = 402$, $p < 0.05$). The different characters represent significant differences among treatments ($p < 0.05$, Tukey-Kramer test). Rest, resting cells; Cont, control; Spx, siphonaxanthin.

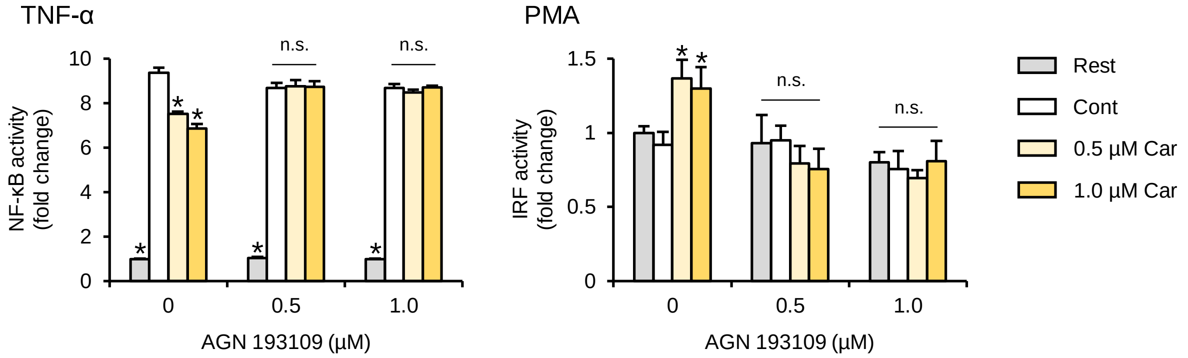

Figure S2. Effects of antagonism of RAR on suppression of TNF-α-NF-κB axis and promotion of PMA-IRF axis by β-carotene. THP1-Dual cells were incubated with 0.5 or 1.0 μM of β-carotene with 0.5 or 1.0 μM of AGN 193109, an antagonist for RAR, followed by stimulation with the indicated agent. β-carotene and AGN 193109 were dispersed in culture medium using DMSO (maximum final concentration, 0.1%) as a vehicle. The resting cell and control groups were incubated for 24 h, followed by no stimulation or stimulation with inflammatory inducer, respectively. After stimulation, SEAP and luciferase activities in the supernatant were measured (*n* = 4). Values represent mean ± SD. Significant differences were detected by one-way ANOVA in TNF-α ($F(11,36) = 1660$, $p < 0.05$), and PMA ($F(11,36) = 13.13$, $p < 0.05$). The asterisks * indicate significant difference from the control group, and n.s. indicates no significance ($p < 0.05$, Tukey-Kramer test). Rest, resting cells; Cont, control; Car, β-carotene.

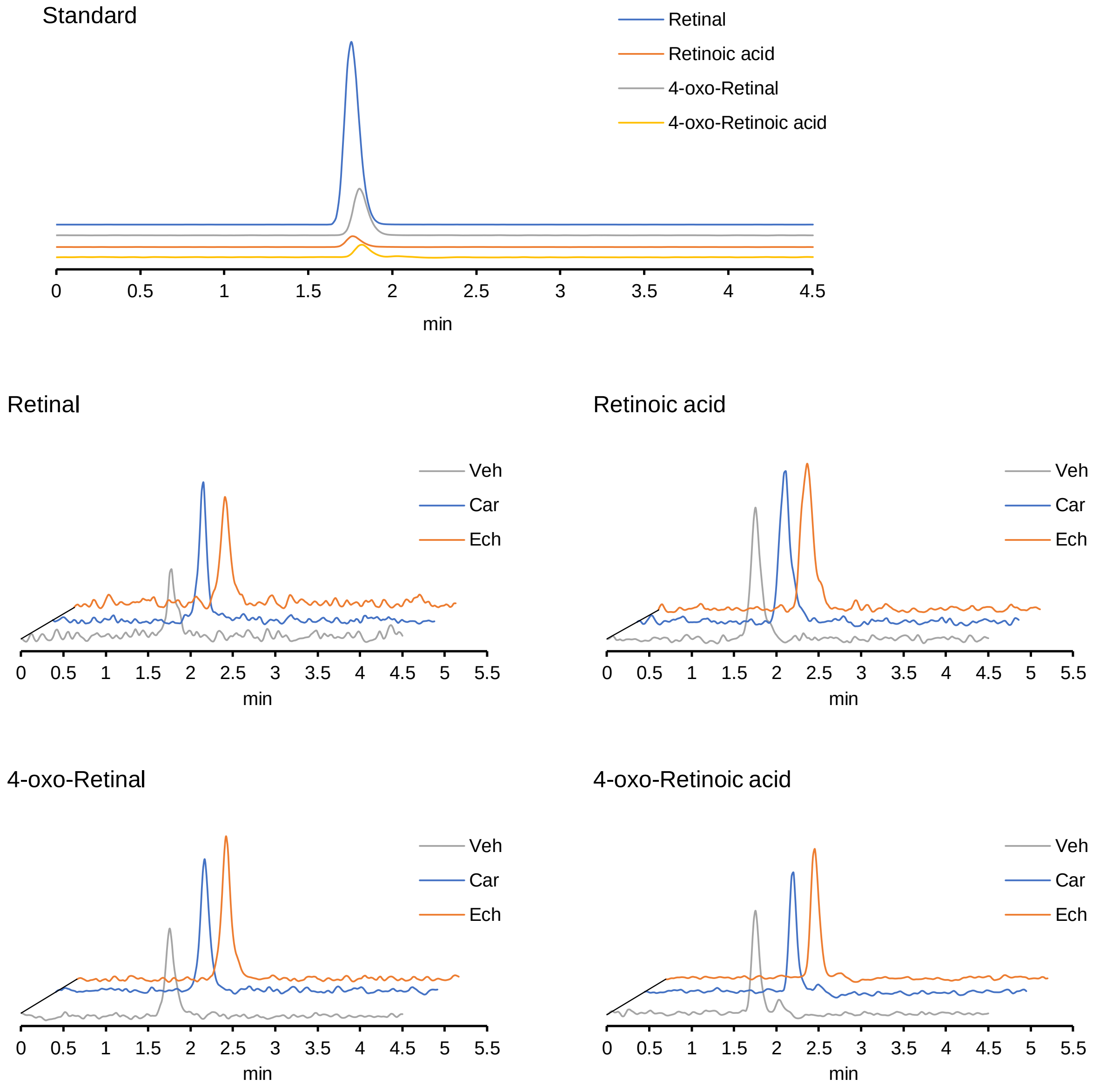

Figure S3. LC-MS/MS analysis of retinoids. Authentic standards and total lipids extracted from the cells were subjected to LC-MS/MS analysis. MRM chromatograms are illustrated.

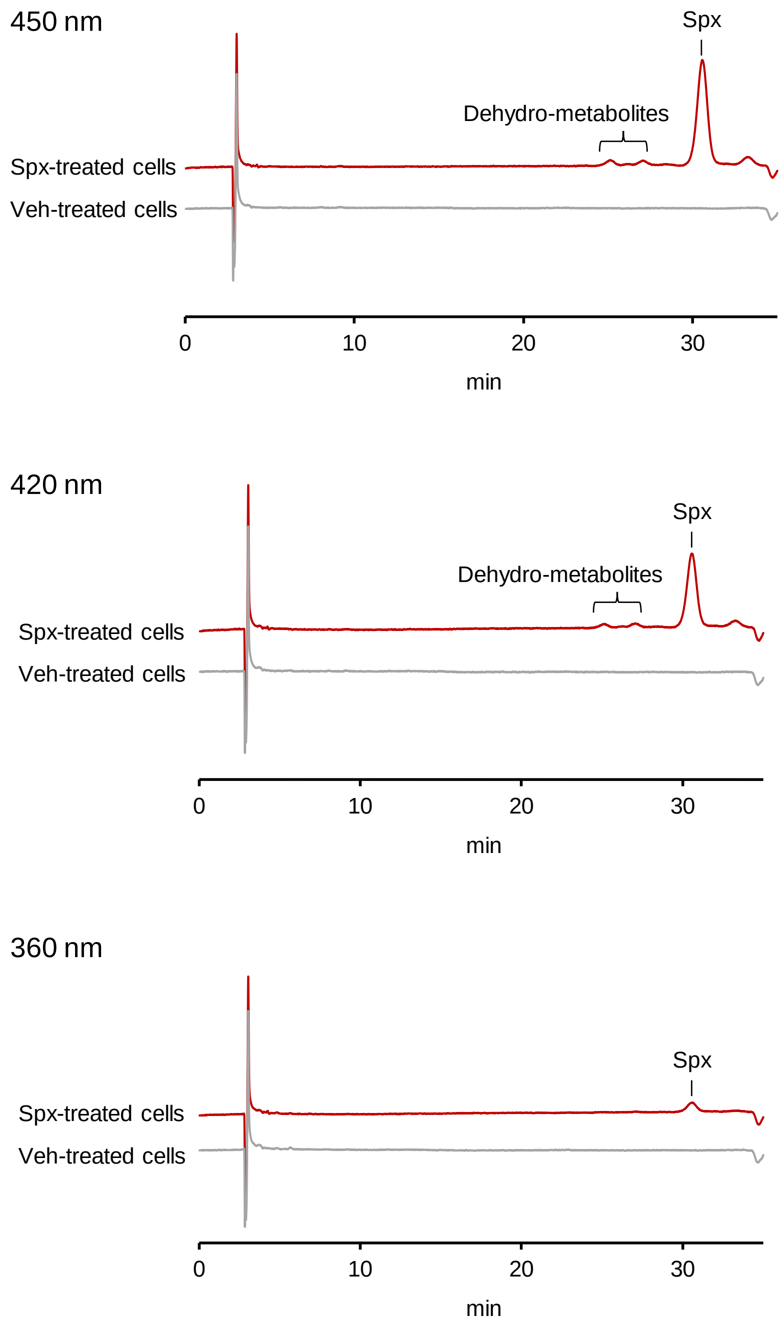

Figure S4. HPLC-PDA analysis of siphonaxanthin and its metabolites. Total lipids extracted from the cells were subjected to HPLC-PDA analysis. Chromatograms at 450 nm, 420 nm, and 360 nm are illustrated.

Table S1. The parameters for the MRM detection in LC-MS analysis of carotenoids and retinoids.

Q1, Q1 m/z; Q3, Q3 m/z; DP, declustering potential (volts); EP, entrance poten-tial (volts); CE, collision energy (volts); CXP, collision cell exit potential (volts).

Target	*Q1*	*Q3*	*DP*	*EP*	*CE*	*CXP*
β-Carotene	536.3	444.4	116	10	21	18
Echinenone	551.3	91.0	156	10	113	12
Zeaxanthin	568.3	91.0	111	10	119	12
Astaxanthin	597.3	147.1	106	10	25	18
Alloxanthin	565.3	91.0	116	10	107	14
Lutein	568.3	77.0	91	10	129	12
Siphonaxanthin	601.3	91.0	101	10	125	12
Fucoxanthin	659.3	109.1	116	10	27	14
Fucoxanthinol	617.3	109.1	136	10	25	14
Retinal	285.0	161.1	76	10	13	14
Retinoic acid	301.1	123.1	106	10	19	16
4-oxo-Retinal	299.1	77.1	111	10	89	12
4-oxo-Retinoic acid	314.8	115.1	111	10	95	14
